# Gas Phase Thermochemistry for Perfluoroalkyl Carboxylic Acids

**DOI:** 10.1021/acs.jpca.5c04296

**Published:** 2025-09-16

**Authors:** Bradley Welch, Narasimhan Loganathan, Angela K. Wilson

**Affiliations:** Department of Chemistry and the MSU Center for PFAS Research, 3078 Michigan State University, East Lansing, Michigan 48824, United States

## Abstract

Perfluorinated species have become ubiquitous due to their desirable industrial properties including thermal and chemical stability, water resistance, and stain resistance. Despite their utility and widespread use, a number of PFAS have been identified as significant environmental contaminants posing health hazards. Much of the current studies are targeted to understand the fate and distribution of PFAS in water and solid interfaces. In contrast, the current insight into the thermodynamic properties of PFAS is minimal but play a critical role for modeling their reactions. In this study, the thermodynamic energies of perfluoroalkanoic acids (C*_n_* F_2*n*+1_OOH) have been predicted. The correlation consistent Composite Approach (ccCA) and coupled-cluster singles, doubles, with triple excitation (CCSD­(T)) methods have been utilized to determine enthalpies of formation and Gibbs free energies including corrections for the conformational ensemble at 298 K. In addition, density fitted and local natural orbital coupled cluster approaches have been used to allow for the evaluation of larger PFAS with the ccCA composite. Thermodynamic predictions made using DFT­(B3LYP) have also been evaluated in comparison to ccCA and CCSD­(T) energetics due to its widespread usage in computing thermochemical properties.

## Graphical abstract

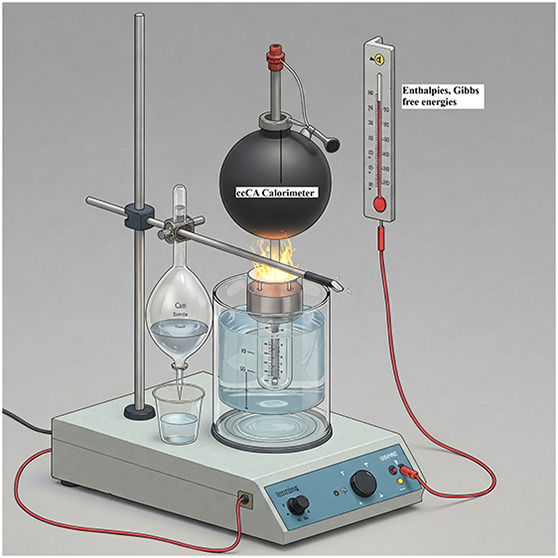

## Introduction

Exposure to per- and polyfluoroalkyl substances (PFAS) is inevitable due to their widespread use for many decades in consumer and commercial applications. The unique amphiphilic properties of PFAS are predominantly responsible for their utility, while the ability of PFAS to resist degradation and bioaccumulate has been linked to a broad range of health impacts (i.e., carcinogenic, autoimmune and developmental disorders, kidney disease, thyroid disease) in humans and animals.^1–7^ The ubiquitous presence of PFAS in natural settings including air, water, soil, and sediments represent some of the most important pathways for PFAS exposure to humans and animals.^8–11^ Consequently, there has been significant progress over the past decade on mitigation strategies to remove PFAS from water by employing different adsorbent materials.^12–14^ As well, there has been an increasing number of studies which are focused on understanding the fate of PFAS in surface soils and sediments under a variety of environmental conditions.^15–18^

Despite the increased use of volatile PFAS molecules both in indoor (e.g., carpets, paints) and outdoor environments (e.g., landfill gas, aqueous film forming foam (AFFF)), there have been limited studies on the behavior of PFAS in air.^8,19–25^ Among the studies, McDevitt et al.^19^ identified the presence of 6:2, 8:2 and 10:2 fluorotelomer alcohols (FTOH) and other PFAS in a number of indoor settings including residences, offices, and carpet stores. Importantly, their study reported that 6:2 FTOH was dominantly present in high concentrations, irrespective of the indoor setting. Similarly, Cahuas et al.^26^ showed that the possible exposure to FTOH in paints increases during the paint drying process, particularly impacting professional painters. In addition, air sampling studies using chemical ionization mass spectroscopic by Riedel et al.^27^ determined the concentration of both 6:2 and 8:2 FTOH in AFFF. Recent studies by Roth et al.^28^ further substantiated the presence of five different FTOH in the gas phase during AFFF usage via gas chromatography coupled with mass spectroscopy, demonstrating the occupational hazard for fire fighters. Most importantly, their studies identified ten different perfluorocarboxylic acids (PFCA) with varying chain length (C_5_–C_16_) and concentrations in AFFF. As well, an increasing number of studies indicate that FTOHs act as precursor molecules for the formation of highly stable PFCA in air and thus can enhance the exposure risk.^8,19,29^ PFCA’s also have been detected in the air of water treatment facilities, and in the flue gas of waste disposal sites.^30–32^ Clearly multiple avenues exist where PFCA’s have transitioned from the aqueous phase to the gas-phase.

The long-range atmospheric transport of PFAS molecules could be one of the primary reasons for their presence in remote regions such as the Artic.^33–36^ A recent review by Evich et al.^8^ highlighted that the atmospheric transport of PFAS and their precursor molecules significantly depends on their molecular properties i.e volatility, molecular weight, and reactivity of the species.^37–39^ Furthermore, PFCA’s have been detected in both atmospheric particulates, as well as in the gas-phase.^40,41^ The transport of PFAS in the atmosphere has a substantial impact on the presence of these species across the globe. While the presence of PFAS in urban areas that are near to industrial or waste management sites is not surprising, more isolated portions of the world can also experience detectable concentrations of PFAS such as far into the interior of Antarctica.^42^ The presence of PFCA’s near coastal regions may be related to ocean currents and sea spray aerosols, in addition to atmospheric transformations of FTOH precursor species. PFAS have also been detected in northern Arctic regions.^43^ The PFAS in the northern Pacific can be explained by ocean currents carrying PFAS from waste streams, though PFAS was also found within ice core samples, suggesting atmospheric deposition. In the Canadian portion of the Arctic, PFAS was found in samples over a 38-year span via ice core sampling.^44^ What makes this unique is that this portion of the Arctic is isolated from potential transport arising from ocean currents. Thus, the PFAS would need to have been deposited via atmospheric sources. PFAS has also been found in isolated regions far from industrial sources including the Antarctic, the Tibetan plateau, and the Sahara desert.^42,45,46^

In considering the role of FTOH’s, FTOH species can react with chlorine and hydroxyl radicals, NOx species, ozone (O3) and Creigee intermediates in the atmosphere to form stable PFCA’s.^47–54^ Studies by Ellis demonstrated that the oxidative transformation of FTOH to PFCA’s can be initiated by the abstraction of hydrogen from the CH_2_ group that is connected to the alcohol group.^47^

The majority of these prior studies have focused on the detection of different types of PFAS molecules in the air and atmosphere. To obtain a more comprehensive understanding of the fate and transport of PFAS, greater thermochemical insight about a broad range of PFAS molecules and their precursors in the gas phase is needed. Such fundamental gas-phase knowledge about PFAS is reported in a limited number of studies.^55–57^ However, most of those studies provide gas-phase data based on determining the Gibbs free energy of legacy PFAS molecules and their isomers.^58–60^ However, a wide array of PFAS have been reported and may be possible in the atmosphere. Many of the current studies employ low-level methods to obtain the Gibbs free energies of PFAS. However, the reported energetics determined using low-level methods are susceptible to high uncertainties due to the nature of methods used in their studies. Thus, a systematic evaluation of thermochemical properties for a larger set of short- and long-chain PFCA’s is necessary especially when sampling studies indicate the existence of very long chain PFCA’s (C ≤ 14).^61^

Importantly, thermochemical energies are necessary for understanding the reactivity of perfluorinated compounds. With suitable choices of methodologies, computational chemistry has been successful in predicting reliable thermochemical quantities for molecules in the gas phase, including enthalpies of formation of organic species.^62^ While numerous computational methodologies can be considered, *ab initio* composite schemes can be particularly effective. They can provide accuracy akin to accuracy that can be achievable with a higher-level *ab initio* methodology, but with greater computational efficiency.

In this work, the thermochemical composite scheme, the *correlation consistent Composite Approach* (ccCA) of the Wilson group,^63–65^ has been used to determine the enthalpies and Gibbs free energies of short- and long-chain PFCA ranging from C_2_ to C_14_ Notably the current study is one of the first to emphasize and obtain thermodynamic quantities with higher-level *ab initio* methods. These approaches are necessary to determine a systematic energetic set – a set of energies that are not subject to dramatically increasingly larger errors in prediction with increasing molecule size – for PFCA’s, which is presently unavailable. A density functional approach is also utilized to demonstrate a number of challenges in considering several commonly utilized density functionals for such sets of molecules.

## Methodology

One of the challenges with PFAS species is that there are many possible conformers. Thus, an important first step in the calculations was to determine the lowest ground-state conformer. For example, for the C_14_ system, there are over 5000 conformers that all have relative energies within a 6 kcal mol^–1^ window relative to the lowest energy conformer. Thus, to obtain the lowest-energy ground-state conformer, the Conformer-Rotamer Ensemble Sampling Tool­(CREST) approach was used.^66,67^ CREST is based upon a metadynamics formalism, providing a highly credible conformational search protocol. CREST made use of GFN2-xTB as the electronic structure method.^68^ The lowest-energy structure from CREST was then optimized with B3YLP using the D3 dispersion correction with Becke-Johnson dampening (D3BJ)^69,70^ with the def2-TZVPP basis set.^71^ Provided within the Supporting Information (SI) is validation that CREST generates the lowest energy conformer. The default settings for CREST were used i.e a 6.0 kcal mol^–1^ window and 0.125 Å RMSD for sampling. Using B3LYP-D3BJ and def2-TZVPP the first few conformers generated by CREST were validated for C_8_–C_12_.

To ensure that the identified structure was a minimum on the potential energy surface, a frequency computation was performed. The frequency calculation provided enthalpic and Gibbs free energy terms to enable the conversion from the 0 K electronic energy to Δ*H*_f__,__298 K_ and Δ*G*_f_,_298 K_. To obtain the entropic term to convert enthalpy to the Gibbs free energy, the quasi-rigid-rotor-harmonic oscillator (qRRHO) approximation in ORCA was used to compute the entropy.^72^ qRRHO modifies low frequency modes that can introduce large errors into the rigid rotor harmonic oscillator model. The contribution from the conformational ensemble computed by the entropy code in CREST^73^ for both the enthalpy of formation as well as Gibbs free energy was used, i.e., the enthalpy and Gibbs free energy that are reported included a correction at 298.15 K for the conformational ensemble. Figure 1 shows the overall workflow to determine thermodynamic quantities for the PFAS molecules considered.

**Figure 1:**
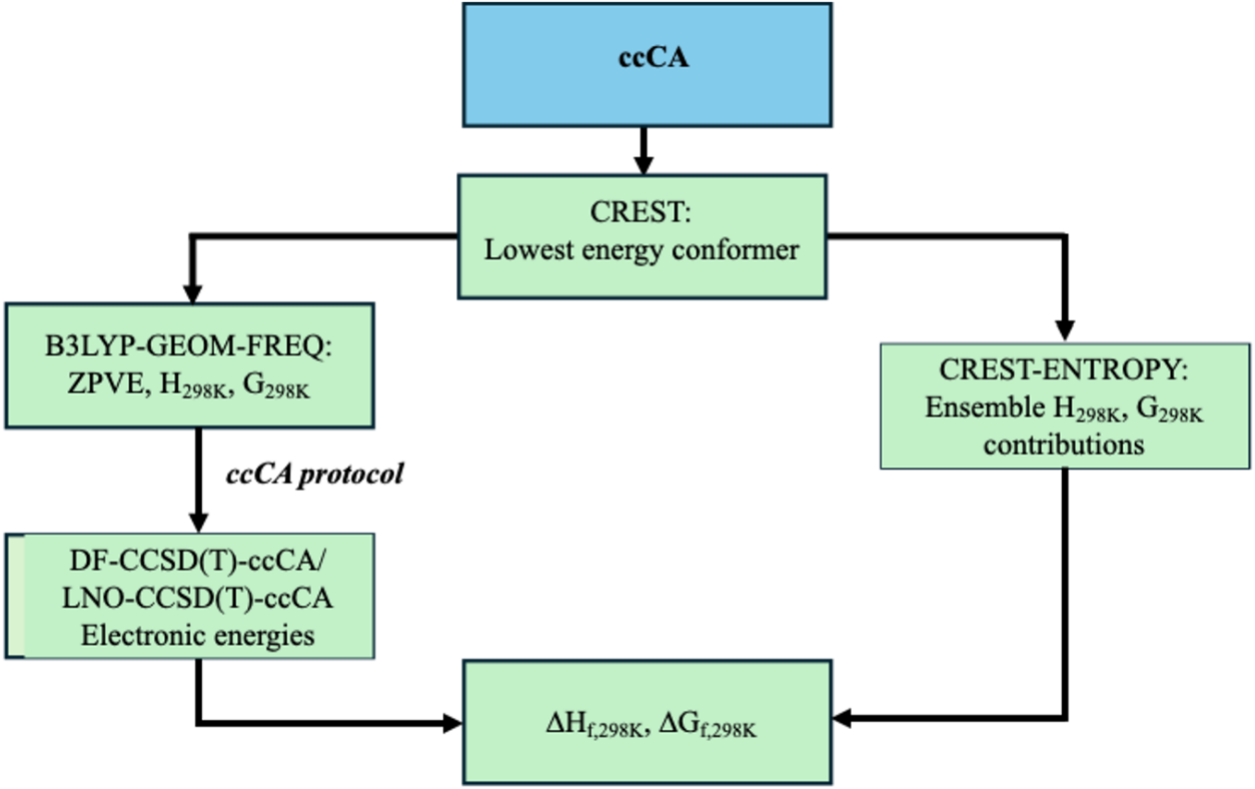
Schematic representation of the workflow used to determine the thermodynamic quantities.

Using the optimized structure, calculations were done to determine the energetic properties using ccCA. The general formulation for the ccCA thermochemical scheme is shown in eq 1, with the implementation based on previous studies^74^ including a composite, RI-ccCA, that utilized density fitting approaches for the MP2 portion of the composite.^75^

Though ccCA has been described previously, herein several different variations to eq 5 below are considered. To put this in context, however, the overall method is described to put into context the modifications that have been considered. Overall, the composite includes contributions from MP2, CCSD­(T), scalar relativistic effects, zero-point energies, spin–orbit corrections, as well as complete basis set (CBS) considerations.

The ccCA composite energy (*E*_ccCA_) is described in eq 1, composed of a number of contributions to the energy

1 $$E_{\mathrm{ccCA}}=E_{\mathrm{ref}}+\Delta E\left(\mathrm{CC}\right)+\Delta E\left(\mathrm{CV}\right)+\Delta E\left(\mathrm{SR}\right)+\Delta E\left(\mathrm{ZPE}\right)+\Delta E\left(\mathrm{SO}\right)$$

Here, *E*_ref_, the reference energy, combines the Hartree–Fock and MP2 correlation energies, each determined at the complete basis set (CBS) limit.

2 $$E_{\mathrm{ref}}=E_{\mathrm{HF}}\left(\mathrm{CBS}\right)+E_{\mathrm{MP}2}\left(\mathrm{CBS}\right)$$

*E*_HF_ is extrapolated to the CBS limit, utilizing the following two-point formula^76,77^

3 $$E\left(n\right)=E_{\mathrm{HF}}\left(\mathrm{CBS}\right)+B\;\exp\left(-1.63n\right)$$

where the two-points utilized refer to energies obtained from Hartree–Fock calculations done subsequently with aug-cc-pVTZ and aug-cc-pVQZ basis sets.^78,79^ *B* is a constant that is obtained from the fit. *n* refers to the cardinal number of the basis set; here, *n* = 3 for aug-cc-pVTZ and *n* = 4 for aug-cc-pVQZ.

The *E*_MP2_(CBS) contribution to *E*_ref_ is obtained by utilizing a three-point extrapolation of a series of MP2 correlation energy contributions (MP2/aug-cc-pVDZ, MP2/aug-cc-pVTZ, and MP2/aug-cc-pVQZ) to the CBS limit using the following inverse Gaussian formula^80^ where *B* and *C* are constants determined in the fit.

4 $$E\left(n\right)=\mathrm{EMP}2\left(\mathrm{CBS}\right)+B\;\exp\left[-(n-1)\right]+C\;\exp\left[-{\left(n-1\right)}^{2}\right]$$

Δ*E*(CC) in eq 1 is the higher-order correlation energy, representing correlation energy beyond that obtained via MP2. For ccCA, this is computed as the difference between CCSD­(T) and MP2 with the cc-pVTZ basis set.

5 ΔE(CC)=E[CCSD(T)/cc‐pVTZ]−E[MP2/cc‐pVTZ]

For this work three different versions of CCSD­(T) were considered: (a) CCSD­(T) without any approximations, as already shown in eq 5

(b) CCSD­(T) with the density fitting (DF) approximation of Nagy and co-workers^81^

6 ΔE(CC)=E[DF‐CCSD(T)/cc‐pVTZ]−E[MP2/cc‐pVTZ]

(c) CCSD­(T) using a reduced cost local natural orbital coupled cluster developed by Nagy and co-workers^82^

7 ΔE(CC)=E[LNO‐CCSD(T)/cc‐pVTZ]−E[MP2/cc‐pVTZ]

The next term in eq 1 is the core–valence (CV) contribution to the ccCA energy. While the frozen-core approximation is typically utilized in *ab initio* calculation with explicit treatment of the valence electrons, the outer core electrons can have a meaningful effect on the energetic description of a molecule, even for first-row main group species. For the present study, this simply entails including the 1s orbitals in the MP2 calculations, which is referred to as frozen core 1 (FC1). The CV contribution is obtained as follows

8 ΔE(CV)=E[MP2(FC1)/aug‐cc‐pCVTZ]−E[MP2/aug‐cc‐pVTZ]

where the core–valence form of the correlation consistent basis sets, aug-cc-pCVTZ^83^ is utilized.

Δ*E*(SR) in eq 1 represents scalar-relativistic effects that are not accounted for using a nonrelativistic Hamiltonian. The scalar-relativistic effects are obtained by utilizing the second-order Douglas Kroll Hess Hamiltonian (DKH2)^84,85^ and the contribution to the composite energy is computed as the difference between DKH2-MP2 and nonrelativistic MP2 with the aug-cc-pVTZ-DK^86^ and aug-cc-pVTZ basis sets.

9 ΔE(SR)=E[DKH2‐MP2/aug‐cc‐pVTZ‐DK]−E[MP2/aug‐cc‐pVTZ]

Δ*E*(ZPE) is the zero-point energy which is determined from a B3LYP-D3BJ/def2-TZVPP frequency calculation. This ZPE is then scaled with 0.9883 ^87^ to correct for deficiencies of the harmonic oscillator approximation. Finally, Δ*E*(SO) provides the spin–orbit contribution for the atomic or molecular species. Here, the spin–orbit of the atoms is determined from the experimental fine structure,^88^ and j-averaged. E is the energy of the atomic level and j is the angular momentum of the level.

10 ΔE(SO)=−∑E(2J+1)/∑(2J+1)

None of the PFCA molecules (C_2_–C_14_) considered here have degenerate electronic ground states, and thus, there is no need to address spin–orbit splitting at the molecular level. Due to the open-shell nature of the atomic species in this work, the Restricted Open Hartree–Fock (ROHF) approach was used for Hartree–Fock, and MP2. UCCSD­(T) as implemented in MOLPRO uses ROHF orbitals but is unrestricted at the UCCSD­(T) level. DF-CCSD­(T) and LNO-CCSD­(T) both made use of ROHF. Tight localization cutoffs for LNO-CCSD­(T) were accessed by the keyword lcorthr = tight.

As noted earlier, to accelerate the composite, density fitting (DF)/resolution-of-the-identify strategies were utilized, for both Hartree–Fock^89^ and MP2 ^90^ computations. As well, DF was used for two of the three coupled cluster routes described earlier, for both approach (b) DF-CCSD­(T), and (c) LNO-CCSD­(T). In terms of the software used for this study, MOLPRO 2022 ^91^ was used for the scalar relativistic, nonrelativistic, and core–valence Hartree–Fock and MP2 computations, as well as CCSD­(T) without any acceleration techniques. For DF-CCSD­(T) and LNO-CCSD­(T) MRCC 2022 was used.^92,93^ The chain-of-spheres approximation (RIJCOSX) implemented in ORCA 5.0.4 ^94^ was used to accelerate the solution of the SCF equations. Tight integration grids are used throughout ORCA via the defgrid3 keyword.

From the ccCA composite energy the enthalpy of formation at 298.15K (Δ*H*_f,298 K_) and Gibbs free energy at 298.15K (Δ*G*_f,298 K_) can be determined,. Several approaches can be used. The most common is to use an atomization reaction approach. An atomization reaction simply is the decomposition of a molecule into its constituent atoms. Consider the hypothetical reaction

$$A_{x}B_{y}C_{z}\to xA+yB+zC$$

The ccCA energy for each of these species is determined based on eq 1. Subsequently, a total atomization energy (TAE) can be obtained the following way

$$\mathrm{TAE}=\left(x\mathrm{EA}+y\mathrm{EB}+z\mathrm{EC}\right)-E\left(A_{x}B_{y}C_{z}\right)$$

Using the TAE, the Δ*H*_f,298 K_ and Δ*G*_f,298 K_ can be obtained as described in ref 95. Other approaches utilized to determine thermochemical energies are reaction-based schemes. A benefit to this type of approach is error cancellation for electronic structure methods; atomization energy approaches involve products which may have substantially different electronic spins from the parent molecule so such approaches can prove challenging for some theoretical approaches if the underlying electronic structure method cannot adequately describe both the atoms and the molecule with similar accuracy.

In addition to the atomization approach, isogyric reaction-based schemes were also considered in this study. Isogyric schemes utilize reactions in which the same number of paired electrons is present on both sides of the equation in the determination of the Δ*H*_f,298 K_ and Δ*G*_f,298 K_. The Wilson group has previously used reaction schemes with the ccCA method for classes of molecules such as organosulfur compounds and hydrocarbons.^96,97^ These reactions were designed, considering known degradation products for PFAS, and represent possible degradation pathways.^98^ These reactions were used previously in a study of a number of fluorinated species by our group.^57^ ISO 1 (isodesmic reaction R1) and ISO 2 (isodesmic reaction R2) are naming conventions utilized here to represent the following

ISO 1 $$C_{n}F_{2n-1}\mathrm{COOH}+\left(n-1\right)F_{2}\to \left(n-1\right){\mathrm{CF}}_{4}+{\mathrm{CO}}_{2}+\mathrm{HF}$$

ISO 2 $$C_{n}F_{2n-1}\mathrm{COOH}+\left(n-2\right)F_{2}\to \left(n-2\right){\mathrm{CF}}_{4}+{\mathrm{CO}}_{2}+{\mathrm{CHF}}_{3}$$

These two isogyric reactions would be considered a CBH-0 scheme within the Connectivity-Based Hierarchy (CBH). CBH provides a systematic way to generate reactions for the determination of thermochemical quantities.^99–101^ The non-PFAS reference species in the isodesmic reactions are reactant and product species that have established, known thermodynamic quantities. Using these thermodynamic quantities and a computed reaction energy, the unknown thermodynamic quantity, in this case enthalpy and Gibbs free energy can be determined. ccCA has previously been used for isodesmic reactions.^102–104^

The experimental enthalpies of formation for CF_4_, CO_2_, HF, and CF_3_H were obtained from the Active Thermochemical Tables (ATcT).^105^ The ATcT provides up-to-date thermochemical values based on an assessment of computational and experimental data over a network of thermochemical reaction cycles, updated when new thermochemical insight is obtained. The reference Δ*G*_f,298 K_ for CF_4_, CO_2_, HF, and CF_3_H are from JANAF.^106^

## Results and Discussion

Thermochemical data for PFCA from C_2_ to C_14_ is obtained in this work and is more reliable than previous work in part, due to two factors: (i) CREST was employed to identify the lowest-energy conformer; and (ii) contributions to the thermodynamics were included from the conformational ensemble (from CREST) at 298 K. While at 0 K there is only one structure, at higher temperatures other conformations can be accessed and be populated. Due to the size of the PFAS considered within, it is imperative to account for all conformational ensembles. Even smaller organic alkanes like pentane can have a contribution to the enthalpy from conformers at 298.15 K of 0.47 kcal mol^–1^.^107^ In Table 1, the enthalpy of formation, of the PFCA’s are provided, determined using three different thermochemical pathways, the atomization approach and the two isogyric approaches.

**Table 1:** Enthalpy of Formation (Δ*H*_298 K_) Computed with CCSD­(T) for Δ*E*(CC) = CCSD­(T)-MP2 in ccCA Using Three Different Thermochemical Schemes^a^

PFCA molecules	ATOM	ISO 1	ISO 2
C_2_	–244.30	–243.49	–244.65
C_3_	–343.10	–341.94	–343.09
C_4_	–440.65	–439.04	–440.19
C_5_	–545.18	–543.13	–544.28
C_6_	–634.77	–632.26	–633.41

a ATOM = atomization energy, ISO 1 = isodesmic reaction 1, and ISO 1 = isodesmic reaction 2. All results are in kcal mol^–1^.

The ccCA energies obtained using CCSD­(T) (Table 1) and DF-CCSD­(T) contributions (Table 2) are in close agreement for both the atomization and isodesmic thermochemical approaches, with differences of less than 1 kcal mol^–1^ for the atomization approach and less than 0.50 kcal mol^–1^ for the isodesmic reactions up to C_6_. The density fitting approximation, which accelerates computations by replacing four-center two-electron integrals into more compact two- and three-center integrals that can be stored in memory rather than written to disk.^108^ It should be noted that DF-CCSD­(T) has been applied in prior studies of dissociation energies and reaction energies. These applications have demonstrated that the DF approximation introduces very small errors in CCSD­(T) calculations,^109^ as also is shown in Tables 1 and 2.

**Table 2:** Enthalpy of Formation (Δ*H*_298 K_) Computed with the ccCA Composite Scheme for PFCA Molecules from Atomization and Isodesmic Reactions^a^

PFCA molecules	ATOM D	ISO 1 D	ISO 2 D	ATOM L	ISO 1 L	ISO 2 L
C_2_	–244.63	–243.51	–244.66	–243.63	–243.43	–244.39
C_3_	–342.97	–341.97	–343.11	–341.96	–341.72	–342.68
C_4_	–440.47	–439.07	–440.21	–440.35	–439.09	–440.05
C_5_	–544.96	–543.16	–544.30	–543.50	–543.18	–544.15
C_6_	–634.50	–632.30	–633.44	–632.68	–632.33	–633.29
C_7_	–731.71	–728.52	–730.25	–729.51	–729.12	–730.08
C_8_	–828.91	–825.92	–827.06	–826.35	–825.93	–826.89
C_9_	–925.85	–922.45	–923.59	–922.91	–922.45	–923.41
C_10_	–1022.12	–1018.02	–1019.16	–1018.80	–1018.01	–1018.97
C_11_	–1120.43	–1115.91	–1117.05	–1115.96	–1115.10	–1116.06
C_12_	–1218.60	–1213.65	–1214.79	–1214.52	–1213.60	–1214.57
C_13_				–1309.90	–1308.91	–1309.88
C_14_				–1410.94	–1409.89	–1410.85

a Two theoretical implementations of coupled cluster for the Δ*E*(CC) contribution of ccCA were evaluated, DF-CCSD­(T) and LCCSD­(T). All results are in kcal mol^–1^. D = DF-CCSD­(T) for the Δ*E*(CC) contribution to ccCA. L = LNO-CCSD­(T) Δ*E*(CC) contribution to ccCA.

Specifically, for C_2_–C_6_, the atomization energies computed using DF-CCSD­(T) approximation can be used within the ccCA composite as a more cost-efficient alternative to CCSD­(T); This allows for ccCA to be applied to the calculation of thermochemical energies of PFAS species of increasing size. Based on the DF-CCSD­(T) results, a gradual divergence occurs between the atomization energy-based values and the isodesmic reaction-based values as the molecule size increases. For the largest DF-CCSD­(T) computation, C_12_, this difference grows to ∼5 kcal mol^–1^. This trend, previously noted in prior studies for systems such as alkanes, can be driven by basis set superposition error (BSSE).^110^ BSSE arises when there is a mismatch in the size of the basis set used for a molecule and those for the atoms, leading to inaccuracies in energies. Reaction-based schemes, like isodesmic schemes, can aid in cancellation (or partial cancellation) of these errors, compensating for the BSSE in a way that may only be possible for an atomization energy approach by using complete basis set extrapolations with very large basis sets. However, such high-level approaches can become computationally expensive for all but the smallest systems. Thus, reaction schemes can be important for determining thermodynamic quantities of larger PFAS species. The divergence in energies becomes most significant for the largest PFAS as shown in Table 3 for the Gibbs free energy. Overall, for larger molecular systems, the reaction-based schemes are preferred over atomization approaches due to their improved error compensation.

**Table 3:** Gibbs Free Energy of Formation (Δ*G*_298 K_) Computed with ccCA Where DF-CCSD­(T) or LNO-CCSD­(T) Have Been Utilized within A Composite Step^a^

PFCA molecules	ATOM D	ISO 1 D	ISO 2 D	ATOM L	ISO 1 L	ISO 2 L
C_2_	–226.75	–224.93	–226.40	–225.76	–225.43	–226.14
C_3_	–316.56	–315.24	–316.13	–315.55	–314.99	–315.70
C_4_	–402.32	–400.40	–401.28	–402.20	–400.59	–401.30
C_5_	–495.96	–493.43	–494.31	–494.49	–493.45	–494.42
C_6_	–575.13	–572.00	–572.89	–573.31	–572.03	–573.61
C_7_	–661.33	–657.01	–657.90	–659.13	–657.10	–657.81
C_8_	–747.81	–742.66	–743.55	–745.24	–743.15	–743.59
C_9_	–833.39	–828.45	–829.34	–830.44	–828.64	–829.33
C_10_	–919.41	–913.58	–914.46	–916.10	–913.56	–914.27
C_11_	–1006.94	–1008.36	–1009.25	–1002.47	–1007.13	–1007.84
C_12_	–1092.31	–1087.25	–1088.14	–1088.23	–1087.20	–1087.91
C_13_				–1174.38	–1171.05	–1171.76
C_14_				–1254.80	–1251.20	–1251.91

a D = DF-CCSD­(T) for the CCSD­(T)-MP2 contribution to ccCA. L = LNO-CCSD­(T) for the CCSD­(T)-MP2 contribution to ccCA. All results are in kcal mol^–1^.

Table 2 also provides ccCA results with LNO-CCSD­(T) for ΔCC. Note, however, the C_14_ results should be treated with some caution. CREST’s entropy program did not result in convergence for C_14_, so this enthalpy and Gibbs free energy do not include contributions from the ensemble. Even for C_4_, this contribution to the enthalpy is already 0.40 kcal mol^–1^, and grows to 2.62 kcal mol^–1^ for the C_13_ species. Overall, the DF-CCSD­(T) and LNO-CCSD­(T) ccCA atomization energies and the isodesmic energies compare very well; the deviations for the atomization energies for the smallest of the molecules are similar to those of Δ*E*(CC) with DF-CCSD­(T). This is an encouraging result for the reduction of computational costs relative to CCSD­(T), as approaches such as the DLPNO have been shown to have errors that increase as the system size increases.^111^ The difference is consistent at ∼1 kcal mol^–1^ for both the isodesmic and atomization energies when the LNO-CCSD­(T)-ccCA-based approach is compared to DF-CCSD­(T)-ccCA results. For C_2_–C_6_, the ccCA enthalpies of formation (Table 2) determined with LNO-CCSD­(T) and the isodesmic reactions are similar to the CCSD­(T) ccCA enthalpies (Table 1) when utilizing the atomization thermochemical approach.

Before analyzing the performance of LNO-CCSD­(T) ccCA for larger molecules, it is useful to evaluate its behavior for C_6_, as this is the largest of the molecules that was treated with CCSD­(T) within the ccCA framework. This allows for a direct comparison with both the canonical CCSD­(T) and more cost-efficient methods. For C_6_, LNO-CCSD­(T) ccCA differs from CCSD­(T) ccCA by 2.09 kcal mol^–1^and from DF-CCSD­(T) ccCA by 1.82 kcal mol^–1^. As the molecule size increases to C_12_, the deviation between DF-CCSD­(T) ccCA and LNO-CCSD­(T) ccCA also increases when using the atomization approach.

Interestingly, thermochemical values obtained from LNO-CCSD­(T) ccCA via the atomization energy approach closely align with those obtained from isodesmic reaction schemes, however, the difference between the DF-CCSD­(T) ccCA and LNO-CCSD­(T) ccCA energies using the atomization energy approach grows with system size. The difference stems from the DE­(CC) contribution to the overall ccCA energy. While both methods result in negative DE­(CC) values, LNO-CCSD­(T) ccCA results in a larger negative energy contribution. The atomic energies calculated by LNO-CCSD­(T) and DF-CCSD­(T) are nearly identical, whereas the molecular energy computed by LNO-CCSD­(T) ccCA is lower. As a result DE­(CC) becomes more negative for LNO-CCSD­(T), leading to lower enthalpies and Gibbs free energies with the atomization energy approach, and the DF-CCSD­(T) ccCA are in better agreement with the energies obtained via isodesmic reactions, particularly for larger PFAS compounds.

Using a local coupled cluster approach (LNO-CCSD­(T)) within ccCA provides significant computational savings relative to a conventional CCSD­(T) approach. Figure 2 illustrates the relative computational times for ccCA using both LCCSD­(T) and DF-CCSD­(T) as alternatives to traditional CCSD­(T). As demonstrated in refs,^112,113^ LCCSD­(T) has enabled calculations on molecules containing tens to hundreds of atoms. While not evident from the graph, for C_6_, LCCSD­(T) requires only 20% of the time needed by DF-CCSD­(T). This efficiency becomes even more pronounced for larger systems – for instance, for C_10_, LCCSD­(T) takes only 2% of the time required for DF-CCSD­(T). These substantial cost savings make LCCSD­(T) within the composite particularly advantageous for studying large PFAS, whereas DF-CCSD­(T) can become computationally intractable.

**Figure 2:**
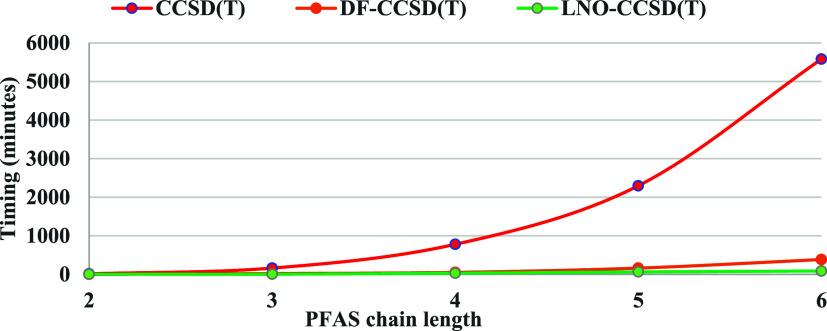
Timing for CCSD­(T), DF-CCSD­(T) and LNO-CCSD­(T) of CCSD­(T)-MP2 in the ccCA composite scheme. Timing is in minutes.

### DFT and CCSD­(T) and DF/LNO-CCSD­(T) Energetics

Table 4 provides the enthalpies of formation calculated using B3LYP-D3BJ/def2-TZVPP alongside energies determined with ccCA (with LNO-CCSD­(T) for the ΔCC contribution), which are provided for comparison. The differences relative to the ccCA enthalpies increase significantly with system size, not only for the atomization energy approach, but also for the isodesmic approaches. Only for the C_2_–C_5_ species do isodesmic reactions 1 and 2 yield enthalpies that are within a few kcal mol^–1^ of the ccCA values.

**Table 4:** Enthalpy of Formation (Δ*H*_298 K_) Computed with B3LYP-D3BJ/def2-TZVPP^a^

PFCA molecules	ATOM	ISO 1	ISO 2	ATOM L	ISO 1 L	ISO 2 L
C_2_	–231.30	–247.60	–242.99	–243.63	–243.43	–244.39
C_3_	–324.95	–348.86	–344.26	–341.96	–341.72	–342.20
C_4_	–417.30	–448.83	–444.23	–440.35	–439.09	–440.05
C_5_	–509.44	–548.59	–543.98	–543.50	–543.18	–544.15
C_6_	–601.43	–648.19	–643.58	–632.68	–632.33	–633.29
C_7_	–693.37	–747.74	–743.14	–729.51	–729.12	–730.08
C_8_	–785.25	–847.24	–842.63	–826.35	–825.93	–826.89
C_9_	–877.33	–946.94	–942.33	–922.91	–922.45	–923.41
C_10_	–969.33	–1046.55	–1041.95	–1018.80	–1018.01	–1018.97
C_11_	–1061.32	–1146.16	–1141.56	–1115.96	–1115.10	–1116.06
C_12_	–1153.91	–1246.37	–1241.76	–1214.52	–1213.60	–1214.57
C_13_	–1245.23	–1345.30	–1340.69	–1309.90	–1308.91	–1309.88
C_14_	–1337.92	–1445.60	–1441.00	–1410.94	–1409.89	–1410.85

a LNO-CCSD­(T) ccCA enthalpies included for comparison. All units in kcal mol^–1^.

These growing deviations are expected, as similar trends of increasing error with system size have been shown for the alkanes in comparing enthalpies determined with B3LYP with those determined by experiment and by CCSD­(T)/large basis set calculations.^63^ Nonetheless, the results are somewhat disappointing. B3LYP, with its relatively low computational cost (formally N^4^ with respect to system size) is appealing, even when compared ccCA-LCCSD­(T) approach -and DFT-based approaches could be appealing for degradation studies of large PFAS species. However, given the observed energy differences, even for reaction-based thermochemical approaches, the ccCA-LNO-CCSD­(T) approach provides a much more robust and reliable strategy for molecules across a broad range of sizes.

A consistent feature of ccCA since its inception has been the inclusion of the Δ*E*(CC) contribution, defined as the energy difference between CCSD­(T) (or, in this work, DF-CCSD­(T) or LNO-CCSD­(T)) and MP2, computed using the modest cc-pVTZ basis set. Given that a CCSD­(T), DF-CCSD­(T), or LNO-CCSD­(T) energy is already available as part of the composite calculation, a natural question arises: how do thermochemical values obtained solely with CCSD­(T)/cc-pVTZ, DF-CCSD­(T)/cc-pVTZ or LNO-CCSD­(T)/cc-pVTZ compare to those of ccCA, DF-CCSD­(T) ccCA, or LNO-CCSD­(T) ccCA?

As shown in Table 5, these single-point calculations exhibit poor agreement with the reference values in Table 1 for the atomization energy approach, even for small systems like C_2_. Although the isodesmic reactions yield better agreement, differences in the predicted energies still increase with molecular size. Notably, even canonical CCSD­(T)/cc-pVTZ – which involves no approximations to reduce computational cost – performs poorly relative to the energies reported in Table 1 for both the atomization energy and isodesmic reaction approaches.

**Table 5:** Enthalpy of Formation (Δ*H*_298 K_) Computed with Single Point CCSD­(T)/cc-pVTZ, DF-CCSD­(T)/cc-pVTZ and LNO-CCSD­(T)/cc-pVTZ^a^

PFCA molecules	ATOM C	ISO 1 C	ISO 2 C	ATOM D	ISO 1 D	ISO 2 D	ATOM L	ISO 1 L	ISO 2 L
C_2_	–218.04	–243.49	–244.65	–217.80	–245.79	–244.48	–217.28	–245.7	–244.21
C_3_	–305.48	–341.94	–342.64	–307.97	–345.49	–344.18	–306.92	–345.24	–343.75
C_4_	–397.50	–439.04	–440.19	–396.89	–443.94	–442.63	–395.72	–443.95	–442.46
C_5_	–486.33	–543.13	–544.28	–485.43	–542.01	–540.70	–483.9	–542.03	–540.54
C_6_	–575.26	–641.07	–639.76	–573.96	–640.07	–638.76	–572.06	–640.11	–638.62

a C = CCSD­(T)/cc-pVTZ. All units in kcal mol^–1^.

It is tempting to assume that isodesmic approaches could provide sufficient error cancellation to allow for reliable predictions from correlated methods with small basis sets. However, this assumption does not hold for the PFCA species examined here. As demonstrated here, even CCSD­(T)/cc-pVTZ performs poorly for the isodesmic approach, and the errors observed with DF-CCSD­(T) and LNO-CCSD­(T) are consistent with this trend. Overall, CCSD­(T), DF-CCSD­(T), or LNO-CCSD­(T) calculations using the cc-pVTZ basis set achieve the accuracy of the composite methods, which incorporate additional components – basis set extrapolation, core–valence contributions, and relativistic effects – which are essential for reliable thermochemical predictions across a range of molecular sizes.

## Conclusions

For the first time, thermodynamic properties for both short- and long-chain perfluoroalkanoic acids (PFCAs, C_2_–C_14_) have been determined by using high-level *ab initio* methods such as ccCA. Importantly, the use of DF-CCSD­(T) and LNO-CCSD­(T) in place of coupled cluster in the ccCA framework, yields reliable thermochemical data with significantly reduced computational cost. The results demonstrate that for smaller PFCAs, the thermochemical quantities from DF-CCSD­(T)- and CCSD­(T)-based ccCA are in excellent agreement. For larger molecules, where canonical CCSD­(T) can become impractical, LNO-CCSD­(T) closely reproduces the atomization and isodesmic reaction energies calculated with DF-CCSD­(T). This demonstrates LNO-CCSD­(T)’s suitability for modeling the thermochemistry of larger PFCA systems and highlights its potential utility for broader application in PFAS reactivity studies. This work also demonstrates that CCSD­(T) calculations with a small basis set do not obtain adequate thermochemical values, even when considering reaction schemes. This also occurs for the widely used B3LYP-D3BJ method. Compared to the composite thermochemical values, this approach results in errors of tens of kcal mol_=_^–1^ even with the isodesmic reaction schemes.

Composite schemes such as ccCA are essential for obtaining credible thermodynamic quantities, especially in the absence of experimental data and can serve as reliable stand-ins until such measurements are available. These accurate values are not only valuable in understanding the gas phase transport of PFAS as well as toward calculating reaction energies and barrier heights. The current study demonstrates that ccCA incorporating DF-CCSD­(T) or LNO-CCSD­(T) can provide reliable energetics for use in modeling atmospheric transformations of PFAS precursor and perfluorinated species. Given its efficiency and accuracy, ccCA can be used to model unknown or hypothesized transformations of these species in atmospheric conditions. Furthermore, accurate thermodynamic date can serve as a critical starting point for understanding PFAS interactions with atmospheric particles – insights that are crucial for predicting how far PFAS species may travel before detachment or deposition, and how they may interact.

## Supplementary Material



## Notes

Relative ordering of the first five CREST conformers optimized with B3LYP-D3JB/def2-TZVPP (PDF)
