# Supplementary material for: Gas Phase Thermochemistry for Perfluoroalkyl Carboxylic Acids
Source: J Phys Chem A. 2025 Sep 16;129(38):8772–82. doi: 10.1021/acs.jpca.5c04296 (PMC12478855; doi:10.1021/acs.jpca.5c04296)
Supplement: Supplementary file 1 [file jp5c04296_si_001.pdf]

Supporting Information for

Gas Phase Thermochemistry for Perfluoroalkyl Carboxylic Acids

*Bradley Welch, Narasimhan Loganathan and Angela K. Wilson**

[*]Department of Chemistry and the MSU Center for PFAS Research, Michigan State University, East Lansing, Michigan 48824, United States

Corresponding author: akwilson@msu.edu

Contains:

Table - 1

Table S1: Relative energy of conformers of the first five conformers from CREST reoptimized with B3LYP-D3BJ. All energies in kcal mol^{-1}.

Conformer/Molecule	C_8	C_9	C_{10}	C_{11}	C_{12}
1	0	0	0	0	0
2	0.22	0.33	0.35	0.33	0.34
3	0.91	0.89	0.13	0.05	0.07
4	0.88	0.51	0.50	0.05	1.75
5	0.75	0.62	0.63	0.49	0.50
